# Discovery and biological confirmation of a highly divergent Tacaribe virus in metatranscriptomic data from neotropical bats

**DOI:** 10.1128/msphere.00520-24

**Published:** 2024-09-11

**Authors:** Carlo Fischer, Murilo Henrique Anzolini Cassiano, William R. Thomas, Liliana M. Dávalos, Yolanda Leon, Jackeline Salazar, Stephen J. Rossiter, Andres Moreira-Soto, Jan Felix Drexler

**Affiliations:** 1 Charité–Universitätsmedizin Berlin, corporate member of Freie Universität Berlin and Humboldt Universität zu Berlin, Institute of Virology, Berlin, Germany; 2 Department of Ecology and Evolution, Stony Brook University, Stony Brook, New York, USA; 3 Consortium for Inter-Disciplinary Environmental Research, Stony Brook University, Stony Brook, New York, USA; 4 Instituto Tecnológico de Santo Domingo, Santo Domingo, Dominican Republic; 5 Universidad Autónoma de Santo Domingo, Santo Domingo, Dominican Republic; 6 School of Biological and Chemical Sciences, Queen Mary University of London, London, United Kingdom; 7 Tropical Disease Research Program, School of Veterinary Medicine, Universidad Nacional, Heredia, Costa Rica; 8 German Centre for Infection Research (DZIF), Charité-Universitätsmedizin Berlin, Berlin, Germany; University of Michigan, Ann Arbor, Michigan, USA

**Keywords:** virology, arenaviruses, Tacaribe, virus evolution, bats, genomics

## Abstract

First isolated from neotropical fruit bats in Trinidad in 1956, Tacaribe virus (TCRV) has rarely been detected since. We searched for New World arenavirus reads in roughly 5.7 million sequencing runs available on public databases using Serratus. We recovered a complete genome of a divergent TCRV in metatranscriptomic data derived from heart and eye tissue of an adult male Jamaican fruit-eating bat sampled in the Dominican Republic, 2014. In total, 2,733 reads were mapped resulting in mean coverages of 7.4-fold for the L and 10.2-fold for the S segment. Re-testing original bat specimens showed the highest viral loads in liver tissue (245 copies/mg). Sanger sequencing of PCR amplicons from liver confirmed correctness of and completed the genome recovered from metatranscriptomic data, revealing conserved arenavirus genomic organization, length, intergenic regions, and genome termini. The newly found TCRV strain tentatively named DOM2014 clustered in a basal sister relationship to all other known TCRV strains with which it shared between 83.3%–86.0% genomic and 91.8%–93.7% translated amino acid sequence identity across protein-coding regions. DOM2014 showed a conserved glycine, proline, proline, threonine (GPPT) nucleoprotein motif, which is essential for TCRV interferon β antagonism. Our data confirm the association of TCRV with the bat genus *Artibeus* put into question by lethal experimental infections and scarce bat-derived TCRV genomic data. Broad genetic diversity and geographic spread require assessments of TCRV strain-associated pathogenicity, particularly for DOM2014 as a highly divergent TCRV strain. Confirmation of genomic database findings by testing original specimens provides robustness to our findings and supports the usefulness of metatranscriptomic studies.

**IMPORTANCE:**

Clade B New World arenaviruses (NWA) include rodent-borne lethal hemorrhagic fever viruses, whereas Tacaribe virus (TCRV) stands out because of its detection in bats and its presumably low zoonotic potential. However, the bat association of TCRV was put into question by lethal experimental neotropical fruit bat infections and rare TCRV detection in bats. Scarce genomic data include near-identical viruses from Caribbean bats and ticks from the US sampled 50 years later. The prototype TCRV isolate used for experimental risk assessments has an extensive passage history in suckling mouse brains. Exploring the true genetic diversity, geographic distribution, and host range of bat-borne NWA is pivotal to assess their zoonotic potential and transmission cycles. We analyzed metatranscriptomic data for evidence of NWA identifying a highly divergent TCRV in bats and confirmed virus detection in original biological materials, supporting the association of TCRV with neotropical bats and warranting investigation of strain-associated TCRV pathogenicity.

## OBSERVATION

New World arenaviruses (NWA) are classified in clades A–D with many clade B members causing lethal hemorrhagic fever in humans (1). Among clade B members, Tacaribe virus (TCRV) is considered non-pathogenic for humans (2). However, accidental laboratory infection with TCRV causing flu-like symptoms in humans has been reported (2). While other known clade B NWA are associated with rodent hosts (1), TCRV was originally isolated from eleven neotropical fruit bats (*Artibeus*) and one mosquito pool between 1956 and 1958 in Trinidad (3). These eleven bats were specified as Jamaican fruit-eating bats (*Artibeus jamaicensis*) and great fruit-eating bats (*Artibeus lituratus*), but sequencing studies suggest they were great fruit-eating bats and flat-faced fruit bats (*Artibeus planirostris*) as Jamaican fruit-eating bats are not found on Trinidad (4). Only a single TCRV isolate termed TRVL-11573 obtained from a great fruit-eating bat and a genomic sequence thereof is available from those pivotal investigations (3). In 2012, a TCRV near-identical to TRVL-11573 was isolated from ticks in Florida, USA (5). In 2022, we identified a divergent TCRV in Brazilian flat-faced fruit bats and great fruit-eating bats and a genetically related previously unknown arenavirus termed Tietê virus (TETV) in Brazilian Seba’s short-tailed bats (*Carollia perspicillata*) (6), suggesting more bat-borne NWA may exist.

The increase in publicly available metatranscriptomic data from high-throughput sequencing (HTS) has allowed uncovering of previously unknown viruses in diverse animals and specimens (7). We searched for evidence of unknown NWA in HTS data from mammalian samples available on public databases using Serratus, an open-science viral discovery platform allowing us to search for viral reads in roughly 5.7 million sequencing runs deposited since 2008 (8).

**FIG 1:**
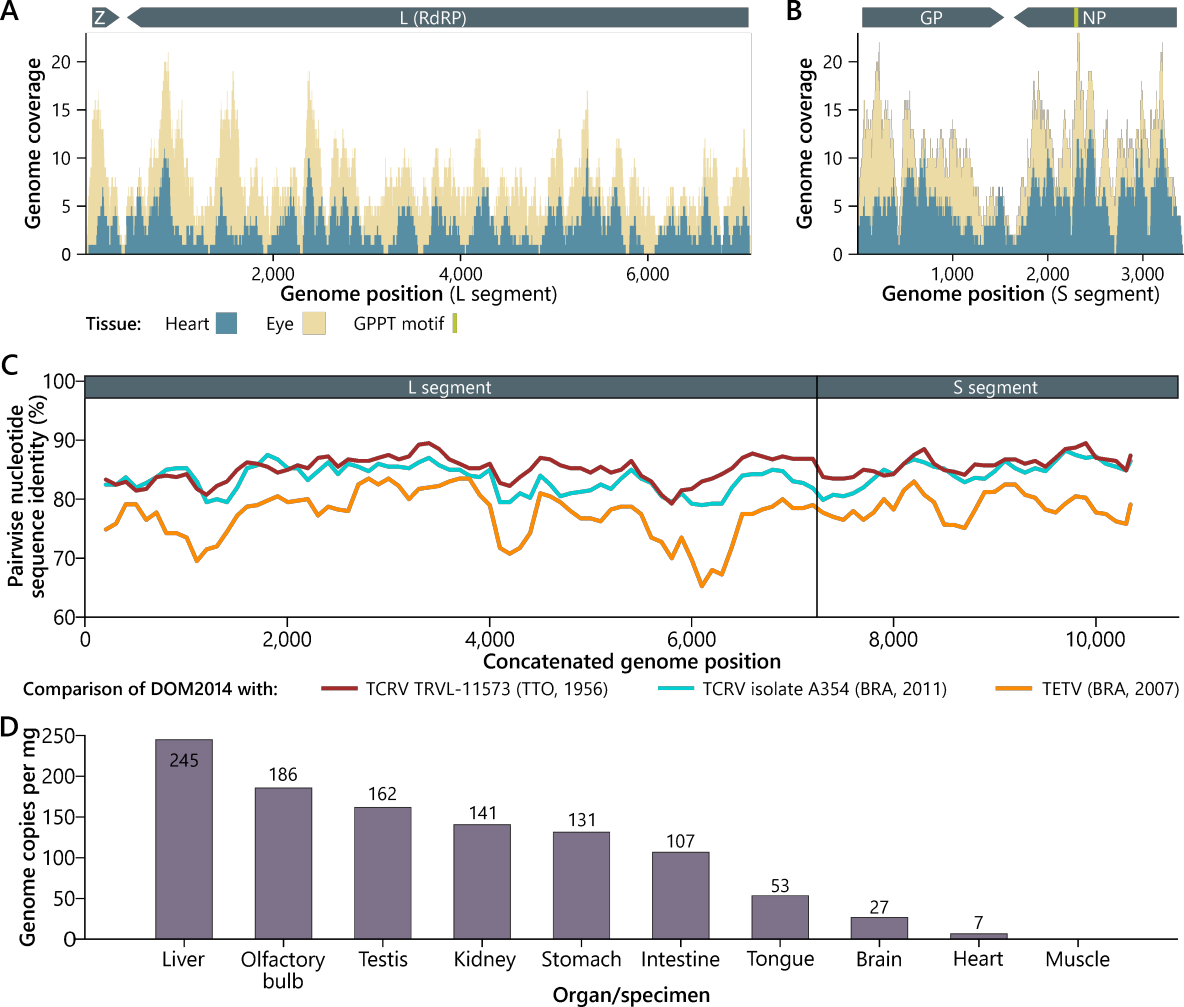
Genome reconstruction and analyses. Genome coverage of TCRV strain DOM2014 compared to the TCRV reference genome for the L (A) and S segments (B). Coding regions and the GPPT motif are indicated. Pairwise genome comparison of DOM2014 with TCRV and TETV (C). L and S segments were concatenated before analyses in SSE version 1.4 (http://www.virus-evolution.org/Downloads/Software/) using a sliding window of 400 bases and an increment of 100 bases. Re-sequenced TCRV strain TRVL-11573 was included, which also represents the almost identical TCRV strain Florida. Organ distribution of TCRV strain DOM2014 (D). Viral copies were determined by real-time RT-PCR. RNA was extracted from approximately 30 mg of each tissue. Bat sampling was approved by the Dominican Ministry of Environment and Natural Resources.

We identified TCRV reads in metatranscriptomic data of heart and eye tissues of one adult, male Jamaican fruit-eating bat (DR011) captured in 2014 at Parque de la Biodiversidad, Autopista Samaná km 51, Monte Plata, Dominican Republic for investigations of bat evolution (9, 10). The complete Sequence Read Archive (SRA) data sets (70 million total reads; SRR9703479 and SRR13417529) were filtered for arenaviral reads using DIAMOND (11) and 2,733 reads were mapped in Geneious to the re-sequenced TCRV TRVL-11573 genome, because the original sequence likely contained several errors (12). The recovered genome was 98.95% complete compared to TRVL-11573 (3). The coverage ranged between 0 and 21 with a mean 7.4-fold for the L segment (Fig. 1A), and 0–23 with a mean 10.2-fold for the S segment (Fig. 1B). Original specimens of the Jamaican fruit-eating bat, which was TCRV infected according to the HTS data were retrieved from archived frozen materials and re-tested. TCRV RNA was detected by strain-specific real-time RT-PCR in brain, heart, intestine, kidney, liver, olfactory bulb, stomach, testis, and tongue tissue, suggesting systemic infection, which is consistent with experimental and natural TCRV infections of bats (2, 6) (Table S1). Viral RNA concentrations were highest in liver tissue with 245 copies per mg (Fig. 1C), whereas overall, viral RNA concentrations were low. To confirm the recovered TCRV genome, 16 conventional and four rapid amplification of cDNA-end amplicons were amplified and sequenced (Table S2). HTS- and Sanger sequencing-derived genomic sequences were fully concordant and allowed recovery of the complete genome of the new TCRV strain tentatively named DOM2014. The TCRV DOM2014 genome was near-equidistant to other NWA sequences across the entire sequence (Fig. 1D). Nucleotide identities were 83.3%–85.0% in the L and 84.5%–86.0% in the S segment compared to TCRV strains TRVL-11573 and A354 recovered by us previously from a wild Brazilian flat-faced fruit bat. The genome organization, intergenic regions, which are essential for transcription termination (13), and complementary genome termini, which likely promote the formation of circular ribonucleoprotein complexes (14) were highly conserved for TCRV strain DOM2014 compared to other TCRV (Fig. S1). Classification of TCRV DOM2014 as a member of the species *Mammarenavirus tacaribeense* was confirmed using the PAirwise Sequence Comparison web tool as recommended by the *Arenaviridae* study group of the International Committee on Taxonomy of Viruses (14). Nucleoprotein (NP) residues 383–407 (including a GPPT sequence motif) that are essential for interferon β antagonism in TCRV (15) were fully conserved in the TCRV strain DOM2014 genome. In contrast, the GPPT motif is replaced by a dysfunctional aspartate, leucine, glutamine, leucine (DLQL) motif in the TCRV prototype (strain TRVL-11573) sequence, potentially due to sequencing artifacts or serial passaging (5). Similarly, the TCRV prototype sequence has a deletion of 12 amino acids in the glycoprotein (GP) compared to contemporary isolates or the re-sequenced prototype virus (5, 12). In TCRV strain DOM2014, this deletion was absent confirming that this deletion is unlikely to occur in TCRV.

**FIG 2:**
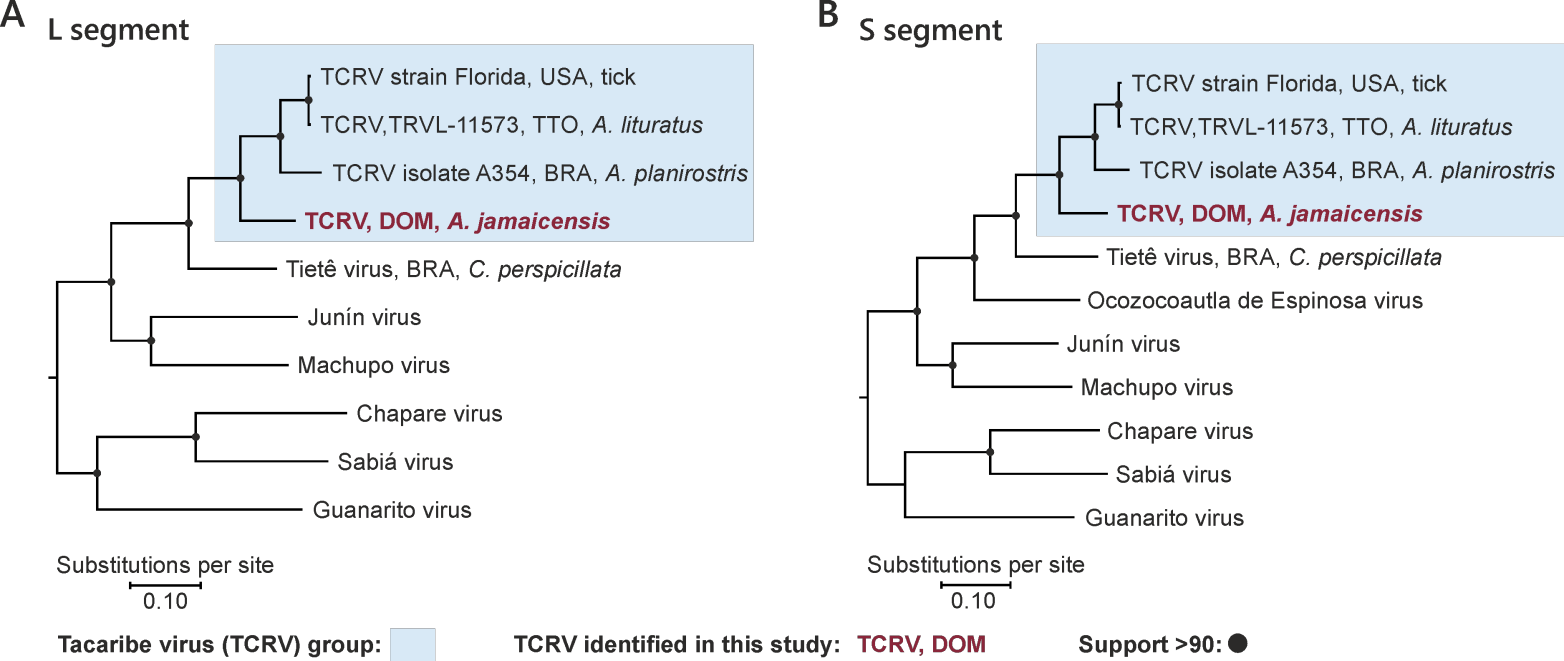
Phylogeny of TCRV strains and genetically related clade B NWA. L segment shown in panel A, S segment shown in panel B. Arenavirus genomes were aligned in MEGA 11 using the MUSCLE algorithm. Maximum likelihood nucleotide sequence-based trees were calculated in MEGA11 using a general time reversible model with uniform substitution rates and a complete deletion option. For each tree, 500 replicates were calculated to provide bootstrap support. Included L and S segment sequences were TCRV strain Florida (MW150032, MW150033), TCRV TRVL-11573 (MT081317, MT081316), TCRV isolate A354 (ON648817, ON648821), TETV (ON648820, ON648824), Ocozocoautla de Espinosa virus (JN897398), Junín virus (NC005080, NC005081), Machupo virus (NC005079, NC005078), Sabiá virus (NC006313, NC006317), Chapare virus (NC010563, NC010562), and Guanarito virus (NC005082, NC005077). Countries are abbreviated according to ISO alpha-3 codes: BRA, Brazil; DOM, Dominican Republic; TTO, Trinidad; USA, United States of America.

In phylogenetic reconstructions of both genomic segments, the newly found TCRV strain DOM2014 clustered at high support in basal sister relationship to the clade containing the flat-faced fruit bat-associated TCRV from our previous field-based study (6), the great fruit-eating bat-associated prototype strain, and the strain obtained from ticks collected in 2012 in the US (Fig. 2). The phylogeny highlighted both broad geographic spread of TCRV and host associations on the bat genus level, confirming the neotropical fruit bat association of TCRV, which was put into question by lethal experimental bat infections (2). Only 3 of the 11 neotropical fruit bats from which TCRV was first isolated were apparently sick and all NWA-infected bat hosts in our recent field study were apparently healthy (6), which is consistent with the ability of bats to maintain transmission of TCRV and related NWA on the population level. Lethal experimental TCRV infections in neotropical fruit bats with the historical TCRV strain TRVL-11573 may be a consequence of viral adaptation as the virus was passaged 20 times in suckling mouse brains. Altogether, the detection of TCRV infections in different neotropical fruit bat species (6) and the detection of antibodies against TCRV in neotropical fruit bats, including short-tailed fruit bats (*Carollia*), and yellow-shouldered bats (*Sturnira*) in Trinidad (16) suggest a broad susceptibility of neotropical leaf-nosed bats (Phyllostomidae) for TCRV and closely related NWA such as TETV.

Among NWA, the capability to enter cells using the human transferrin receptor 1 (TfR1) is a proxy for the potential to cause human disease (17, 18). The prototypic TCRV which was passaged extensively in mice lacked the capability of using human TfR1 (17). Considering that small alterations in the TCRV GP may facilitate efficient usage of human TfR1 and that a single amino acid exchange in the human TfR1 allowed cellular entry of the TCRV prototype strain (17), the zoonotic potential of contemporary TCRV strains deserves investigation. This is also highlighted by the ability of TCRV to cause lethal infections in interferon-deficient mice similar to human-pathogenic clade B NWA members such as Junín virus (19, 20). Immediate experimental assessments may exploit newly available viral isolates from ticks (5) and reverse genetics systems (17) in the case of the divergent TCRV sequences described in our study and previously from Brazilian bats (6). Comparative *in vitro* assessments should include different TfR1 orthologs from bat NWA hosts.

Limitations of our study include the inability to isolate TCRV. However, molecular detection of TCRV in different organs suggests that our data are robust. Systematic analyses of HTS datasets generated from animal hosts provide a useful tool beyond field studies to uncover yet unknown pathogenic arenaviruses in areas of high biodiversity and poor surveillance (1). A major limitation of retrospective analyses of metatranscriptomic or metagenomic data is the lack of independent sequence confirmation. In contrast to many virological data mining projects (7, 8, 21–24), and similar to a recent coronavirus study (25), we re-tested original specimens, confirming our findings and showcased how the combination of *in silico* and *in vitro* methods can uncover viral diversity in archived materials.

## Data Availability

The complete genome segments of TCRV DOM2014 can be accessed on GenBank (PP967231 and PP967232).

## SUPPLEMENTAL MATERIAL

Supplemental material msphere.00520-24-s0001.pdf Supplemental tables and figure.

ASM does not own the copyrights to Supplemental Material that may be linked to, or accessed through, an article. The authors have granted ASM a non-exclusive, world-wide license to publish the Supplemental Material files. Please contact the corresponding author directly for reuse.
