# Supplementary material for: Discovery and biological confirmation of a highly divergent Tacaribe virus in metatranscriptomic data from neotropical bats
Source: mSphere. 2024 Sep 11;9(10):e00520-24. doi: 10.1128/msphere.00520-24 (PMC11520282; doi:10.1128/msphere.00520-24)
Supplement: Supplemental material — Supplemental tables and figure. [file msphere.00520-24-s0001.pdf]

Supplements

Table S1. Primer for TCRV quantifiction by real-time RT-PCR.

Oligonucleotide	Sequence
TCRV_DR11_rtF	AGTTGAGACATGTRTAYCTCATAGA
TCRV_DR11_rtF	ACTTTATGTWGGKGATCTCAACAC
TCRV_DR11_rtP	FAM-CCAACTGCC/ZEN/TCTGCAAAGTCTTCRACTAA-3IABkFQ

Real-time RT-PCRs were conducted using SuperScript™ III One-Step RT-PCR System (Invitrogen). Amplification included 55°C for 15 minutes, 95°C for 3 minutes and 45 cycles of 95°C for 15 seconds and 58°C for 30 seconds with fluorescence read at the 58° annealing/extension step on a LightCycler 480 thermocycler (Roche, Basel, Switzerland). The probe was labeled with fluorescein amidite (FAM). 3IABkFQ, 3' Iowa Black® FQ quencher; ZEN, ZEN quencher.

Table S2. Primers for genome amplification and Sanger sequencing.

**Primer**	**Sequence**	**Amplicon size** (bases)
TCRV_DR11_L1_F1	GATCCTAGGCGTTACGTGCA	542
TCRV_DR11_L1_R1	TACAGCAAATCCCTGAAAGACC	
TCRV_DR11_L2_F1	GATTTCACTCAATGTCTTCCACA	676
TCRV_DR11_L2_R1	TCGAATTACAGGTGGTGGATG	
TCRV_DR11_L3_F1	CATTTGTTTCCACAAGGTCTGC	715
TCRV_DR11_L3_R1	AGTGACTGTGCCATGGCACA	
TCRV_DR11_L4_F1	ATGACTTGAACCATGAAGTTGTAAC	776
TCRV_DR11_L4_R1	TAATCAAGACACTGCAGTCCAG	
TCRV_DR11_L5_F1	GGAAACCACCTTCACCATCTC	726
TCRV_DR11_L5_R1	TGACCAAATTTGTTGCTGCAGC	
TCRV_DR11_L6_F1	ATCAGCTGATTCACCCTTTGAG	811
TCRV_DR11_L6_R1	TGGCAGTGAACAATGGTGACC	
TCRV_DR11_L7_F1	TCAGCAACATTTACTGGAACTTCT	808
TCRV_DR11_L7_R1	TTGCTTCAGACTTGTACAAAGAAC	
TCRV_DR11_L8_F1	CGCCTCACTAGTGAAACCAAG	786
TCRV_DR11_L8_R1	GAGCTAAGGAACGACCCATTC	
TCRV_DR11_L9_F1	TACCTCCCCTTTGTTTTGAACCT	802
TCRV_DR11_L9_R1	TGTGTGAGGAGATGACAACTTG	
TCRV_DR11_L10_F1	TTCAGACCCAGACATGCACTC	835
TCRV_DR11_L10_R1	TACGCTGGTCAATGACAGGG	
TCRV_DR11_L11_F1	AGCAGAGTATCTCATAATGGGTC	854
TCRV_DR11_L11_R1	ACATTGAGGATCAAGAGTATCAAC	
TCRV_DR11_L12_F1	CTGACATCACCTTTAACTCCAAC	704
TCRV_DR11_L12_R1	CGGCACTTGACCATGGATGA	
TCRV_DR11_3RaceL_F1	GAGTGGGACATGGGATTCCA	277
TCRV_DR11_3RaceL_F2	GTCGCTGAGGATCACATCAAC	255
TCRV_DR11_5RaceL_R1	GTTTCTTAACATGATTTGGTGACATC	241
TCRV_DR11_5RaceL_R2	GATAGTGGTCGCTACAGGTG	236
TCRV_DR11_S1_F1	GGATCCTAGGCATTTCTTGTC	730
TCRV_DR11_S1_R1	ATAAGGCACCGTTCACCCTC	
TCRV_DR11_S2_F1	ACTGAGGGCAAACTGATTCAGA	757
TCRV_DR11_S2_R1	AAGTAATTGGGGTTTTCCCCTG	
TCRV_DR11_S3_F1	ACACACTTTCAGGGCAGCAC	710
TCRV_DR11_S3_R1	GCAAGTGTGGGACAAATTCGG	
TCRV_DR11_S4_F1	TCTGGGTAGTTCCCCACTCA	771
TCRV_DR11_S4_R1	GGCAGTAGGAACCCCTACG	
TCRV_DR11_S5_F1	TTTGTCCCTTTCTTTCTGCTGG	608
TCRV_DR11_S5_R1	TGGAATTGTCAGAGTTTGGGATG	
TCRV_DR11_S6_F1	CATTGTTCATGGTCTCCCCTC	642
TCRV_DR11_S6_R1	GATCCTAGGCAAATTGTCTAACTC	
TCRV_DR11_3RaceS_F1	TTCTTTTTGCCTTTCTAAGCACTC	249
TCRV_DR11_3RaceS_F2	CTAAGCACTCGTTGAACCTGAG	235
TCRV_DR11_5RaceS_R1	CGAACAGGATCTGCCAGCC	192
TCRV_DR11_5RaceS_R2	CACACCTGTATAGGTTCACCA	182

PCR amplification was conducted using SuperScript™ III One-Step RT-PCR System (Invitrogen). Amplification included 55°C for 15 minutes, 95°C for 3 minutes and 45 cycles of 95°C for 15 seconds, 58°C for 20 seconds, and 72°C for 45 seconds. Amplification of 5’ and 3’ cDNA ends was conducted using the 5′/3′ RACE Kit, 2nd Generation (Roche Diagnostics GmbH, Germany) according to the manufacturers instructions.

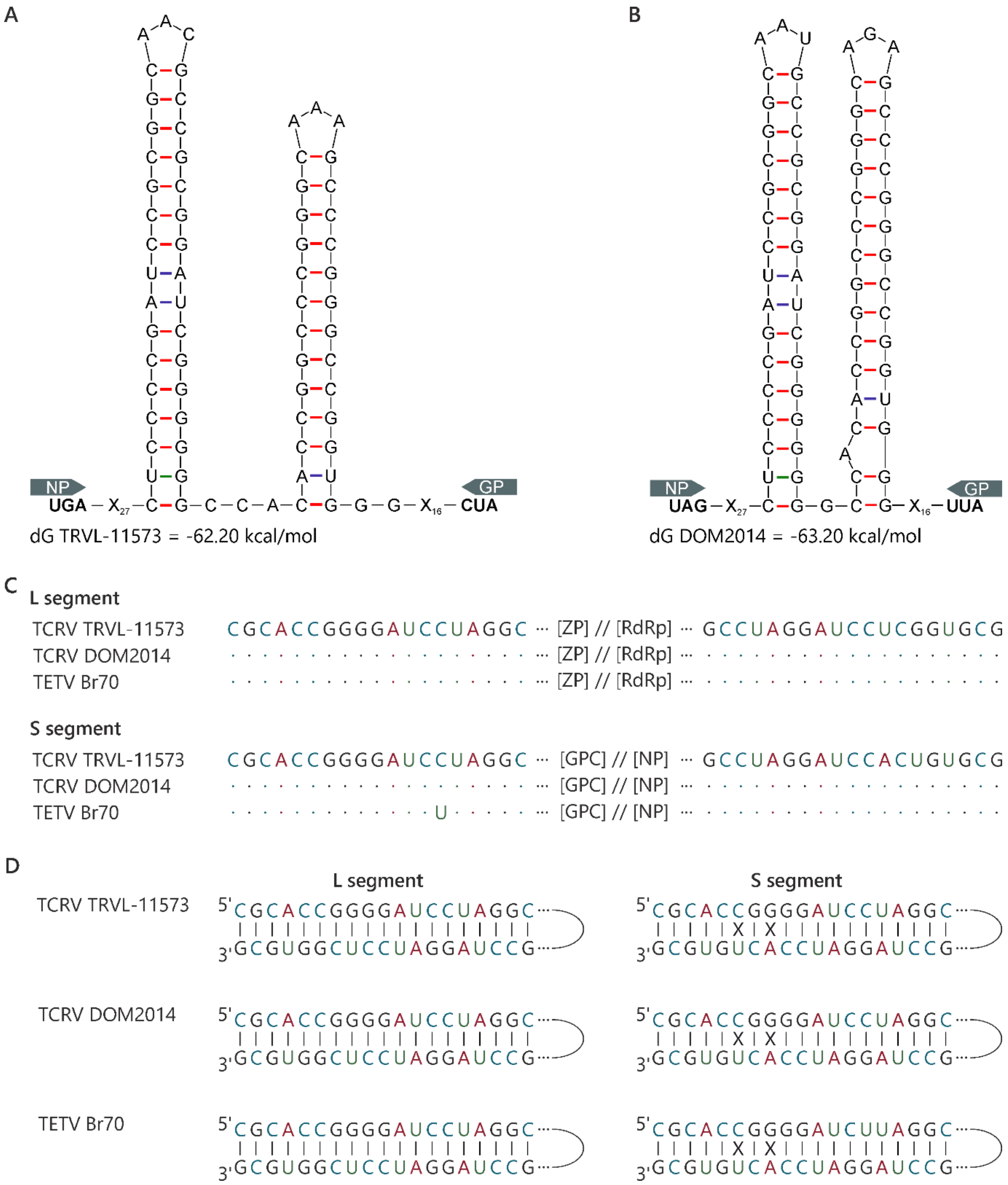

Figure S1. Conserved genomic features. Secondary structure of the S segment intergenomic region for the re-sequenced reference strain TRVL-11573 (**A**) and TCRV DOM2014 (**B**). Not shown bases are indicated by X. Conservation of TCRV strain DOM2014 genome termini (**C**) and complementarity of the 5′- and 3′-untranslated regions (**D**).
